# Neurologic Dysfunction Associated With Mechanically Assisted Crevice Corrosion and Elevated Cobalt Ion Levels After Total Hip Arthroplasty

**DOI:** 10.1016/j.artd.2021.09.002

**Published:** 2021-10-07

**Authors:** Brandon W. Yan, Stefano A. Bini

**Affiliations:** a School of Medicine, University of California San Francisco (UCSF), San Francisco, CA, USA; b Department of Orthopaedic Surgery, UCSF School of Medicine, San Francisco, CA, USA

**Keywords:** Adverse local tissue reactions, Mechanically assisted crevice corrosion, Cobalt toxicity, Neurologic dysfunction, Total hip arthroplasty, Surgical complication

## Abstract

Adverse local tissue reactions secondary to mechanically assisted crevice corrosion (MACC) at the trunnion is a complication of total hip arthroplasty known to cause local soft-tissue damage. However, what is not as well appreciated is that MACC in metal-on-polyethylene (MOP) articulations can lead to cobalt ion serum elevations with associated neurological dysfunction just as in metal-on-metal articulations. We report a compelling case for the association of neurologic dysfunction tied to metal ion elevations secondary to MACC at two distinct MOP tapers in a 58-year-old intensive care unit nurse with two hips implanted 3 years apart. This report further raises awareness about the potential of MACC-generated elevated ion levels to produce neurological symptoms that might otherwise be overlooked in patients with MOP articulations.

## Introduction

Mechanically assisted crevice corrosion (MACC) at the trunnion refers to confirmed wear and corrosion of the femoral head-neck taper in total hip arthroplasty (THA) implants and is sometimes referred to as trunnionosis, taperosis, or pseudotumor [1]. The condition accounts for up to 3% of THA revisions, yet the underlying etiology is imperfectly understood [2,3]. Current hypotheses include wear between metal-on-metal and metal-on-polyethylene modular junctions leading to the release of metal ions from fretting corrosion that is potentially exacerbated by the increasing use of larger femoral heads on narrower tapers with offset necks as well as other specific implant-specific design factors [2,[4], [5], [6]]. Mixed metal-alloy hip prostheses have been implicated in particular [7].

MACC can manifest clinically as adverse local tissue reactions such as implant failure, soft-tissue damage, and, less frequently, end-organ damage [2,4]. Systemic neuropathic manifestations from fretting at the head-neck taper have only been reported in a few cases, [[8], [9], [10]] although numerous cases have documented neurologic dysfunction, such as headaches, hearing loss, tinnitus, vision changes, and polyneuropathy, from other articulations such as metal-on-metal [3,[11], [12], [13], [14], [15], [16], [17], [18], [19], [20]]. Among cases traced to the trunnion, a myriad of systemic symptoms including depression, cognitive deficits, weight gain, hemolytic anemia, sensorimotor dysfunction, decreased appetite, and weight loss have been reported [[8], [9], [10]]. However, a causal relationship between trunnion wear and these nonspecific neurologic symptoms has been hard to establish as these case reports generally report single and somewhat different events for each separate patient.

Toxicity from cobalt ions released into the circulation from total hip replacement implants has been implicated as a mechanism for systemic symptoms [21]. It is postulated that mechanical and oxidative stresses on the prosthetic joint cause cobalt ion release, and the lymphatic and vascular systems subsequently facilitate systemic spread and cytotoxicity [21]. Of the eighteen reported cases of cobalt toxicity from THAs identified in a prior review article, ten involved mental-on-metal articulations, and none were reported as deriving from the trunnion [21]. Thirteen reported neurotoxic manifestations such as paresthesia, tinnitus, and visual loss [21]. It is worth noting that although metal ion toxicity is usually associated with cobalt-chromium implants, chromium is generally considered a minor contributor to toxicity for several reasons including higher threshold of serum concentration for toxicity, precipitation into cobalt phosphate compounds (ie, loss of free chromium ions), and relative insolubility [11,22].

In this case report, we describe the case of a 58-year-old female who underwent staged bilateral THA procedures 3 years apart with the same implants. She subsequently presented with neurologic symptoms associated with elevations in cobalt ion levels caused by MACC at the trunnion 7-8 years after each index surgery.

## Case history

Informed patient consent was taken from the patient. Institutional review board approval was not required as all data are deidentified.

**Figure 1:**
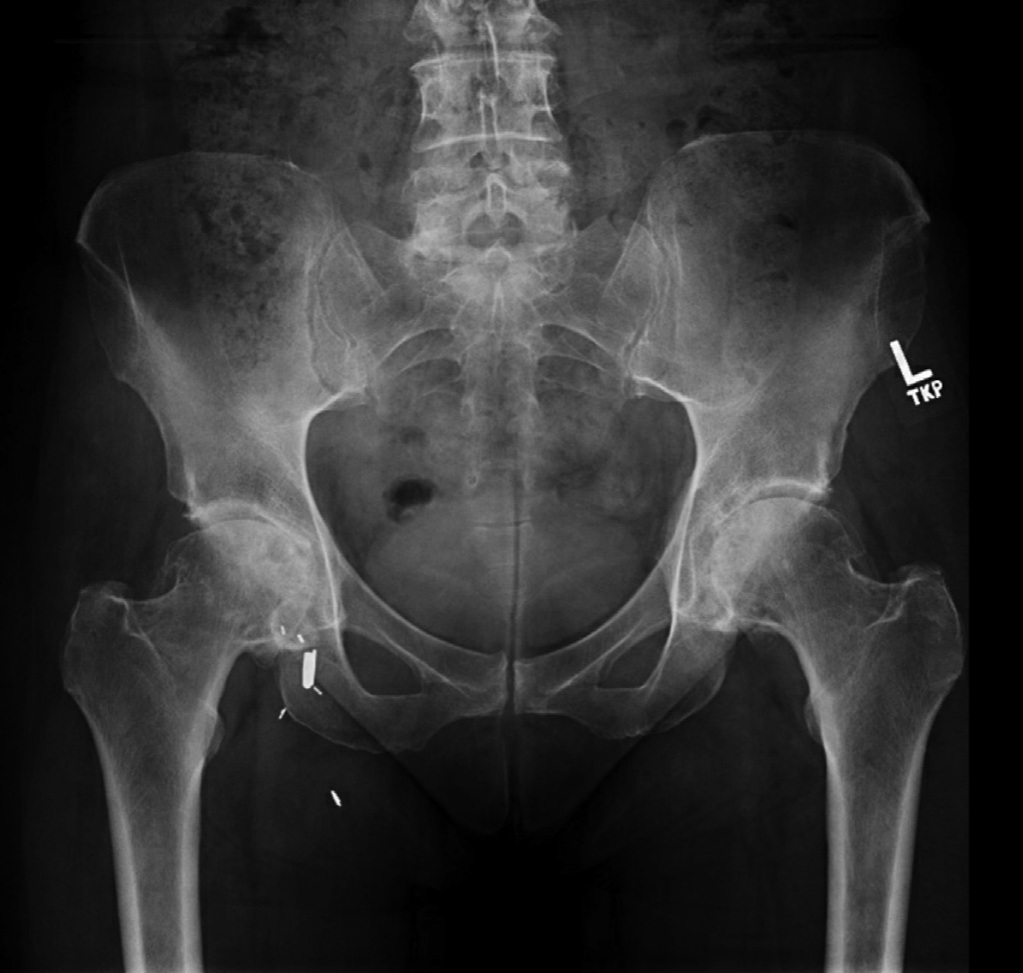
Preoperative pelvic radiograph 2 months after first-stage L-THA, February 2010. Note: Metallic objects seen in the right groin are surgical clips from a remote saphenous vein ligation procedure performed in 1988.

A 58-year-old woman who works as a pediatric intensive care unit nurse at a large academic medical center presented in 2009 with bilateral hip pain and pelvic radiographs consistent with advanced degenerative arthritis of both hips. Her past medical history was notable for longstanding hypothyroidism managed with levothyroxine. A corticosteroid injection was given with temporary relief in each hip. One year later, the hip pain worsened and was significantly impacting her quality of life. Pelvic radiographs demonstrated progression of her degenerative arthritis (Fig. 1).

She subsequently underwent a left total hip arthroplasty (L-THA) in April 2010 at the age of 59 years. An anterolateral approach was used along with an intraoperative repair of the gluteus medius. The following implants were placed: Zimmer Trilogy Cluster-Holed Shell (OD 54 mm; Tivanium Alloy) acetabular cup, Zimmer Longevity Crosslinked Polyethylene acetabular liner (32 mm ID, Standard Lip), Zimmer Versys Cobalt-chromium femoral head (32 mm, +0 mm), and Zimmer ML Taper Standard Offset, size 12.5 mm, Tivanium Alloy femoral stem (Zimmer Biomet Inc, Warsaw, IN). The only complication was a unicortical calcar fracture above the level of the lesser trochanter that was immediately identified and addressed by passing a single cerclage wire around the proximal femur to prevent subsidence.

**Figure 2:**
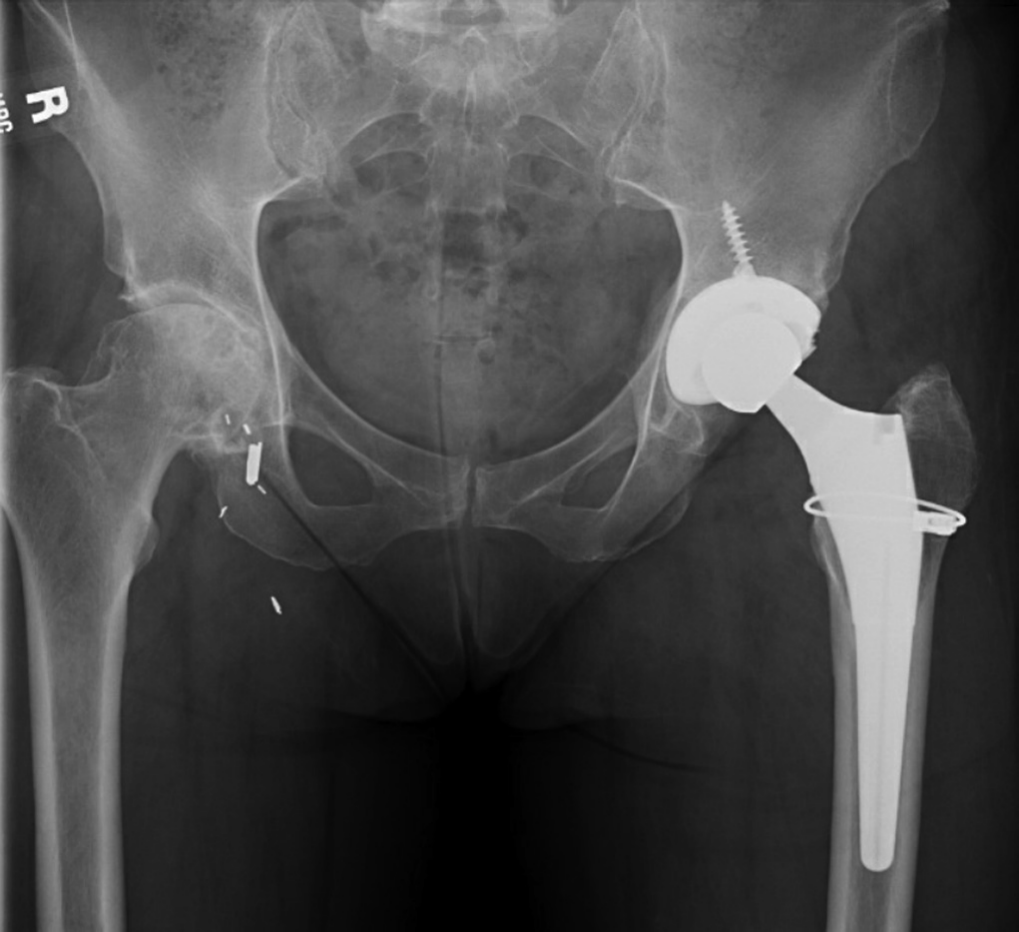
Pelvic radiograph 2 years after first-stage L-THA and 7 months before R-THA, October 2012.

The patient was asked to use crutches for weight-bearing for 6 weeks and had an unremarkable postoperative recovery with excellent osseous integration of the stem and no evidence of subsidence. At the 2-year follow-up, she expressed feeling very pleased with the result of her L-THA and had returned to an active lifestyle. While she reported no significant left-sided hip pain, her right hip pain had grown progressively more painful in association with radiographic evidence of osteoarthritis progression (Fig. 2).

In May 2013, the now 62-year-old patient underwent an uncomplicated right total hip arthroplasty (R-THA) using an anterolateral approach and gluteus medius repair with the following implants: Zimmer Trilogy Cluster-Holed Shell (OD 54 mm, Tivanium Alloy) acetabular cup, Zimmer Longevity Crosslinked Polyethylene acetabular liner (32 mm ID, Standard Lip), Zimmer Versys Cobalt-chromium femoral head (32 mm, −3.5 mm), and Zimmer ML Taper Standard Offset, stem size 12.5 mm, Tivanium Alloy femoral stem (Zimmer Biomet Inc, Warsaw, IN). There were no intraoperative fractures, and the patient was allowed to weight-bear fully.

**Figure 3:**
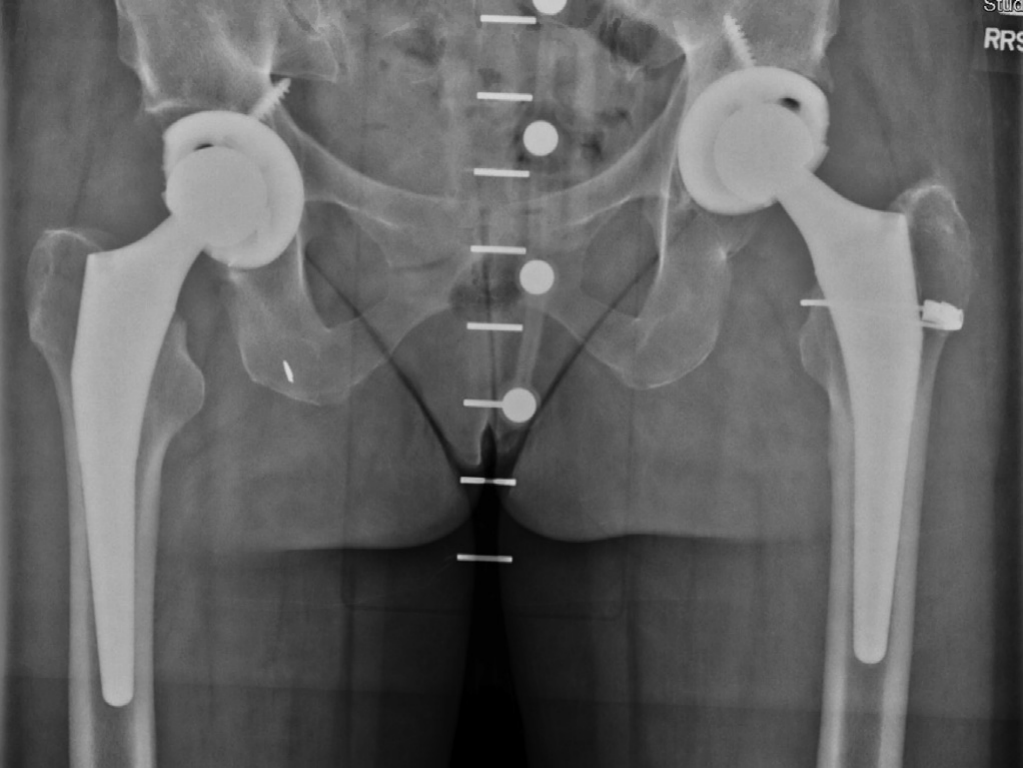
Postoperative pelvic radiograph after staged bilateral total hip arthroplasty, July 2013.

Her postoperative course was uncomplicated, and she was able to walk 2 miles and swim daily by 6 weeks after surgery. Pelvic films at follow-up demonstrated well-fixed and well-aligned cementless bilateral total hip replacements (Fig. 3). She returned to work as a pediatric intensive care unit nurse.

She remained in her usual state of health until May 2015, 5 years after L-THA and 2 years after R-THA. At that point, she reported anterior left hip and groin discomfort. Inflammatory labs showed a normal erythrocyte sedimentation rate (ESR) of 16 mm/h and mildly elevated C-reactive protein (CRP) of 12.7 mg/L. She was referred to physical therapy for hip strengthening. Metal ion levels were not obtained by the treating surgeon.

**Figure 4:**
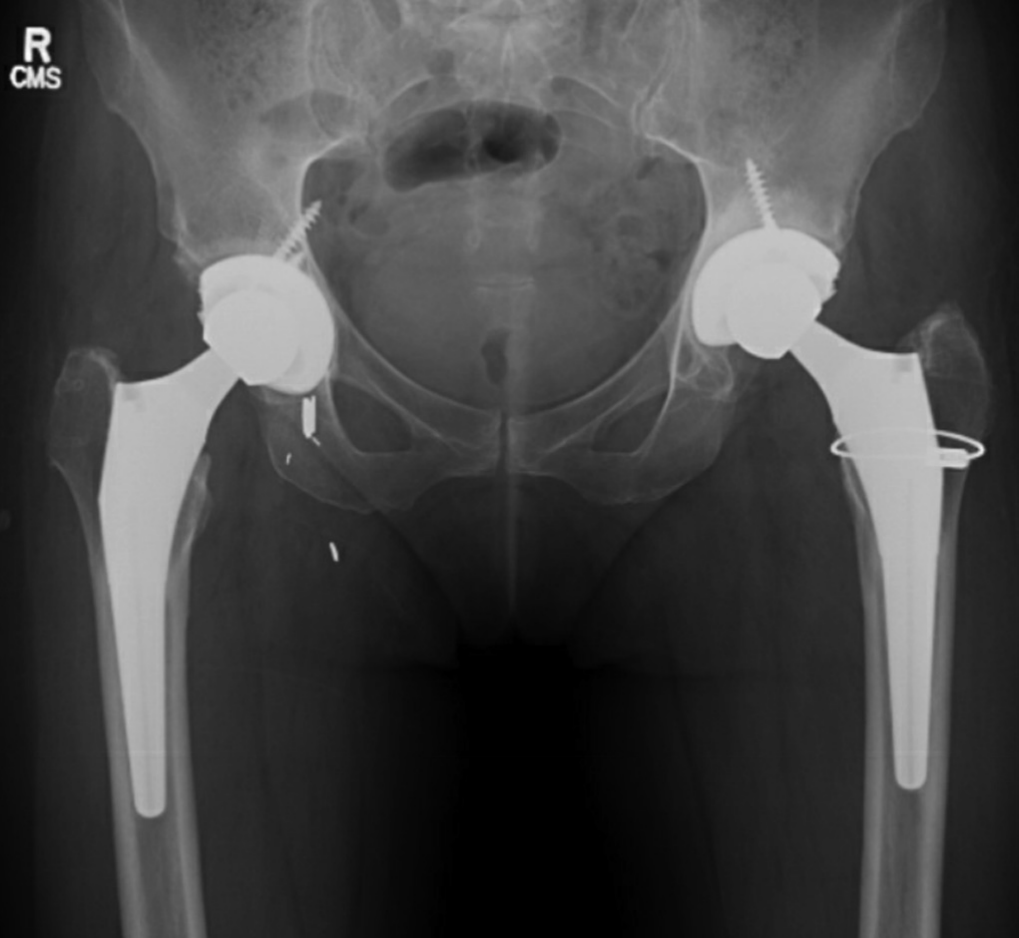
Postoperative pelvic radiograph 7 years after first-stage L-THA, March 2017.

In September 2016, the patient presented with intermittent left-sided posterior gluteal pain associated with strenuous physical activity. Given the infrequency of the pain and onset only with high levels of activity, no further workup was performed, and the plan was to continue monitoring. However, the left-sided intermittent hip pain did not subside. Furthermore, by May 2017, the patient also reported 3 months of new-onset vertigo, headache, generalized fatigue, vision blurriness, and memory issues as well as anxiety and mood disturbance. In particular, the patient described her mood symptoms as a “constant feeling of unrest” and feeling “sad, uneasy, and withdrawn.” Her neurological complaints were alarming and superseded in importance her buttock and groin pain. Now 7 years after L-THA and 4 years after R-THA surgery, metal ion labs were ordered. The results were notable for an elevated plasma chromium level of 4.0 mcg/L and elevated blood cobalt level of 10.6 mcg/L. ESR and CRP were normal. Plain radiographs taken in March 2017 were suggestive of left-sided cup loosening (Fig. 4), and a subsequent multiacquisition variable-resonance image combination protocol magnetic resonance imaging (MRI) of the left hip in May showed a fluid collection consistent with MACC around the implant, as well as evidence of partial gluteus medius tear/detachment along the anterolateral border of the left trochanter.

Given her clinical symptoms and high suspicion for systemic cobalt toxicity from possible MACC, likely gluteus medius deficiency, and possible aseptic loosening of the acetabulum, a L-THA revision surgery was performed in June 2017. Surgical exploration of the L-THA was notable for a loose cup with fibrous ingrowth, black debris inside the 32-mm head and on the femoral neck taper consistent with MACC, and a less than 25% partial detachment of the gluteus medius most likely from a failed repair of the gluteus medius following the anterolateral approach. The extent of metal ion deposition inside the head and on the trunnion was not noted in the record. The cable was removed simply because it served no function and was in no way in contact with the stem. The cup, liner, and femoral head were exchanged with a new cup, polyethylene liner, and ceramic head with a titanium sleeve. The stem was retained, the trunnion manually cleared of gross debris with a wet sponge and no abrasive surfaces, and the gluteus medius tendon tear repaired with a suture anchor using our previously published technique [23]. The patient was asked to protect the gluteal repair by using a walker for 8 weeks after surgery which secondarily protected the cup to ensure ingrowth.

At her 4-week follow-up from the L-THA revision, the patient reported resolution of her neurologic and mood symptoms. At 6 months after L-THA revision, her plasma chromium and blood cobalt levels were within normal limits (2.3 mcg/L and 1.8 mcg/L, respectively).

The patient remained in good health and returned to an active lifestyle for the next 2 years until January 2021, when she presented with 2 months of headaches, vertigo, fatigue, vision blurriness, and memory issues accompanied by anxiety and mood disturbance. The patient reported that these symptoms were similar in nature to those that appeared before her L-THA revision surgery. She had no significant mechanical symptoms or pain in the right hip. Now 8 years after R-THA and 3 years after L-THA revision surgery, labs showed an elevated plasma chromium of 8.1 mcg/L and elevated blood cobalt of 9.4 mcg/L. ESR and CRP labs were within normal limits at 6 mm/h and 1.2 mg/L, respectively.

Metallosis at the trunnion of the original R-THA was suspected, and revision R-THA was recommended. A multiacquisition variable-resonance image combination protocol MRI showed no evidence of a pseudotumor or of a possible gluteus medius failed repair. Notably, the onset of symptoms for the left and right hips both occurred approximately 7 years after THA.

**Figure 5:**
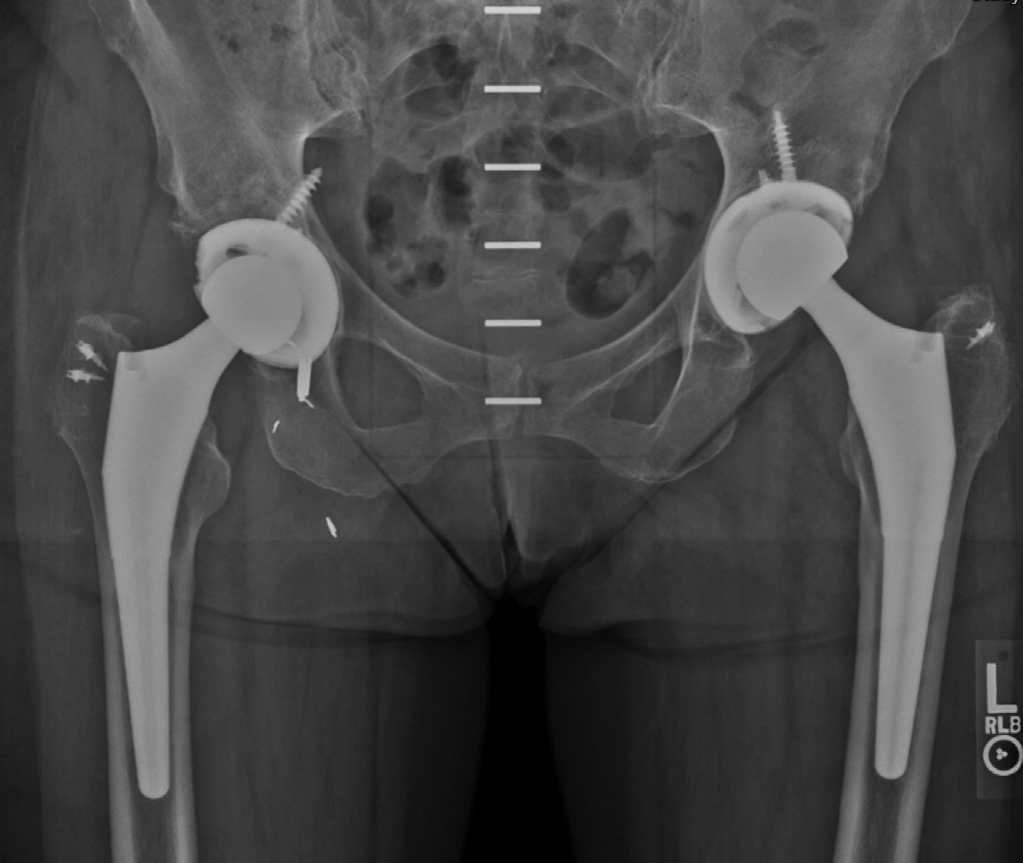
Postoperative pelvic radiograph after bilateral revision total hip arthroplasty, March 2021.

As with the left hip, during the revision R-THA surgery to replace the metal head in February 2021, the trunnion was found to be corroded with deposition of black material inside the head and over approximately 50% of the trunnion. The cobalt-chromium head was replaced with a ceramic head with a titanium sleeve. The cup was well fixed, but a 50% detachment of the gluteus medius tendon from the greater trochanter not seen in the preoperative MRI was seen intraoperatively and surgically repaired [23]. The patient had an uncomplicated postoperative course and was sent home with a walker to protect her gluteus medius repair for 8 weeks. She subsequently resumed full weight-bearing and normal activities and reported resolution of her headaches, memory issues, and other neurologic symptoms by 3 months after the revision. Chromium and cobalt levels obtained at that visit, 2.5 mcg/L and 1.8 mcg/L, respectively, returned to normal limits. Pelvic films showed a well-fixed, well-aligned cementless bilateral THA (Fig. 5). She resumed an active, unrestricted lifestyle and clinical nursing.

## Discussion

This case illustrates a presentation of cobalt toxicity manifesting as neurologic symptoms in the context of MACC at the trunnion after THA. Although fatigue, headache, vertigo, memory decline, and mood changes are relatively nonspecific, the elevated serum cobalt levels on laboratory testing in the absence of other contributory comorbidities or lab abnormalities point to MACC as the most likely cause. In particular, the patient presented twice with similar symptoms, and subsequent workup revealed elevated metal ion levels on a nearly identical timeline after each THA surgery. Resolution of symptoms and ion level abnormalities after revision further supports an association between the patient’s symptoms and MACC. The delayed diagnosis after the initial presentation underlines the poorly understood relationship between the neurologic presentation of cobaltism and THA.

With few case reports in the literature, the existence of systemic cobaltism from MACC at the trunnion, much less its long-term effects, remains poorly established [6,21]. This case provides compelling evidence of the phenomenon and an example of how it could manifest in neurologic symptoms. Of 13 case reports of neurologic symptoms from THA-related cobalt toxicity identified in a prior review, none have occurred with cobalt levels below 20 mcg/L previously [21]. This case suggests that elevated cobalt levels as low as 9.4 mcg/L could be sufficient to cause neurologic symptoms.

Although systemic cobaltism from MACC is exceedingly rare, cobalt toxicity from metal-on-metal contact at the acetabular femoral head interface, occupational exposure, and following treatment of anemia is better described [6,21,24]. For instance, in metal workers exposed to cobalt dust and dissolved cobalt, memory loss, neuropathy, and decreased visual acuity have been reported [8]. Mechanistically, cobalt’s neurologic toxicity can be attributed to its impairment of mitochondrial metabolism leading to cellular dysfunction and death [25]. A systematic review of reported cases of systemic arthroprosthetic cobaltism found a 72%, 52%, and 48% rate of constitutional, audiovestibular, and peripheral motor sensory symptoms, respectively [25]. Thus, the fatigue, vertigo, headache, and memory loss experienced by this patient are consistent with known manifestations of systemic cobalt toxicity.

MACC should be considered in patients with cobalt-containing prosthetic joints presenting with new-onset neurologic changes. The expected onset of symptoms has been reported as being on the order of 1-9 years from implantation with a mean of 3.7 to 4.3 years, [4,12] as MACC results from chronic progressive micromotion at the metal interface. As our case illustrates, symptoms and elevated cobalt levels normalize after THA revision. Other symptoms of MACC and cobalt toxicity include hip pain, adverse local tissue reactions, lower extremity edema, cardiac symptoms, or changes to vision or hearing [4,5]. Standard workup includes focused history and physical examination, laboratory tests for inflammatory markers and serum metal ion levels, consideration of joint aspiration and fluid analysis, and pelvic imaging [4,13,26]. For symptomatic trunnionosis, revision THA is the treatment of choice [4,26].

The recognition of MACC with cobalt implants led to device recalls and a shift in surgical practice to favor ceramic femoral heads, which appear to be less susceptible to corrosion than cobalt-chromium alloy heads [4,12,21]. However, MACC in patients with ceramic femoral heads in THA is still considered possible, and ceramic heads with titanium sleeves are favored [27].

It should be noted that the Zimmer Versys cobalt chromium head implant used in this patient has been implicated in multiple cases of MACC when paired with the Zimmer M/L Taper trunnion [4,6,12]. There is ongoing litigation related to toxicity from the Zimmer Versys cobalt-chromium head and M/L Taper Hip Prosthesis [28].

This case report has several limitations, the most apparent being the lack of generalizability with this research design. The patient’s neurologic symptoms (headache, vertigo, vision blurriness, memory loss) are nonspecific and could be due to alternative causes other than cobalt ion level elevation. As described previously, however, the dual nearly identical presentations, time course, laboratory and surgical finding of MACC during revision surgery, as well as symptom resolution after revision offer an unusually compelling narrative for MACC at the head-neck taper as opposed to other causes. Given the patient’s age, some of these symptoms such as memory loss might be attributed to normal cognitive changes, but age-related dementia would not be expected to reverse with surgical intervention.

## Conclusions

In summary, we presented the case of a single patient who received bilateral, staged, identical THAs and who later developed neurologic symptoms associated with serum metal ion elevations on two separate occasions separated by the same time delay from the index surgeries. We feel this case report makes a compelling argument for a causal link between MACC and systemic cobalt ion toxicity manifesting as neurologic symptoms. This case report alerts clinicians to consider screening for MACC when new-onset neurologic symptoms follow total hip replacement surgery, particularly in patients in whom this type of metal-on-polyethylene device has been implanted.

## Supplementary data

Conflict of Interest Statement for Yan

Conflict of Interest Statement for Bini
