# Supplementary figures and images for: Neurologic Dysfunction Associated With Mechanically Assisted Crevice Corrosion and Elevated Cobalt Ion Levels After Total Hip Arthroplasty
Source: Arthroplast Today. 2021 Oct 7;11:217–21. doi: 10.1016/j.artd.2021.09.002 (PMC8501461; doi:10.1016/j.artd.2021.09.002)

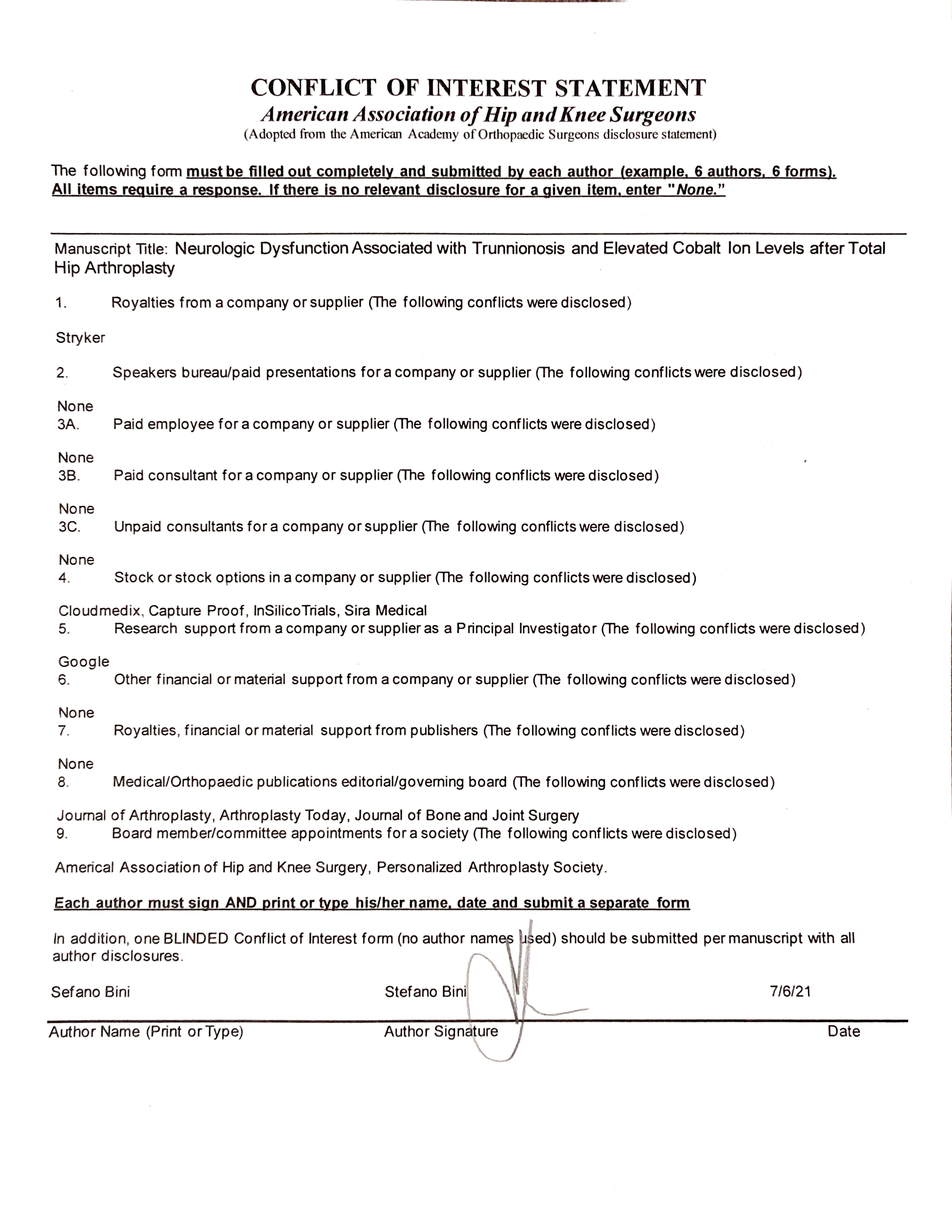

Supplement: Conflict of Interest Statement for Bini [file figs1.jpg]
